# Chiropractic at the crossroads or are we just going around in circles?

**DOI:** 10.1186/2045-709X-19-11

**Published:** 2011-05-21

**Authors:** John W Reggars

**Affiliations:** 1 Suite1/593 Whitehorse Road, Mitcham, Victoria, 3132 Australia; 2 School of Chiropractic and Sports Science, Faculty of Health Sciences, Murdoch University. Murdoch, Western Australia, 6150

## Abstract

**Background:**

Chiropractic in Australia has seen many changes over the past 30 years. Some of these changes have advanced the professional status of chiropractic, improved undergraduate training and paved the way for a research culture. Unfortunately, other changes or lack of changes, have hindered the growth, public utilisation and professional standing of chiropractic in Australia. This article explores what influences have impacted on the credibility, advancement and public utilisation of chiropractic in Australia.

**Discussion:**

The 1970's and 1980's saw a dramatic change within the chiropractic profession in Australia. With the advent of government regulation, came government funded teaching institutions, quality research and increased public acceptance and utilisation of chiropractic services. However, since that time the profession appears to have taken a backward step, which in the author's opinion, is directly linked to a shift by sections of the profession to the fundamentalist approach to chiropractic and the vertebral subluxation complex. The abandonment, by some groups, of a scientific and evidenced based approach to practice for one founded on ideological dogma is beginning to take its toll.

**Summary:**

The future of chiropractic in Australia is at a crossroads. For the profession to move forward it must base its future on science and not ideological dogma. The push by some for it to become a unique and all encompassing alternative system of healthcare is both misguided and irrational.

## Background

This article is based on the inaugural FG Roberts Memorial Lecture, delivered by the author at the Annual Conference of the Chiropractic & Osteopathic College of Australasia, Melbourne, Australia 2010.

Frederick George Roberts was a natural health practitioner embracing chiropractic, osteopathy and naturopathy, who operated many clinics throughout Australia during the 1950's and 1960's. He visited all these clinics regularly and gave public lectures on natural healing. From 1959, his interest turned more to chiropractic and osteopathy and he founded the Chiropractic and Osteopathic College of Australasia in 1959.

This paper is the author's perception of the many changes which have impacted, both positively and negatively, on chiropractic and the chiropractic profession over the past 30 years. Some of those changes have advanced the professional status of chiropractic, improved undergraduate training and paved the way for a research culture. Unfortunately, other changes, or lack of changes, have adversely affected the profession's growth, credibility and the public utilisation of chiropractic services in Australia. It would also appear, that the crossroads confronting the profession in Australia are not unique, as there are many parallels with what has occurred or is occurring internationally.

## Discussion

### Introduction

In order to appreciate and fully comprehend what I perceive to be the crossroads currently confronting the chiropractic profession in Australia, it is necessary to reflect on where the profession has come from, and the roads it has taken to reach these crossroads.

Furthermore, this is not first time the literature has referred to chiropractic being at some sort of crossroads. Others have also opined on similar circumstances facing the profession [1-3]. These three references will be discussed in this article, in the context of the current status of chiropractic in Australia.

### Pre-registration

The author graduated from the Chiropractic College of Australasia (Melbourne) in 1972, and at that time, in all but one Australian State or Territory, chiropractic was not afforded any formal recognition by governments and there was no regulation or registration of chiropractors or osteopaths. Also, at that time, at least within the field of chiropractic, little to no research was being undertaken, and that which was being conducted, was of dubious quality.

As a young student I, like most of my peers, believed without question what I was told by my lecturers, what was written in scholarly journals, and even more so, what was written in books about chiropractic. However, there were some theories that tested the bounds of credibility and were difficult to accept as fact. For example, Fred Stoner in his book "The eclectic approach to chiropractic" [4], discusses the theory of "Vivaxis", which refers to a relationship between the geographical location where one is born and the earth's energy fields. Stoner cites the work of Goodheart, founder of Applied Kinesiology, and writes: *"Dr. Goodheart noted that the direction in which the patient faces may affect the strength of his muscles. After examining over 500 patients, he found that if a patient faces toward the town in which he was born, a definite increase in muscle strength can be noted (as demonstrated through muscle testing). Furthermore, the body often seems to lunge forward when faced toward this direction."*

While to any reasonable person the theory of "Vivaxis" is absurd, it is no more fanciful than some of the contemporary and accepted diagnostic and therapeutic protocols used by many chiropractors today, in the detection and correction of the vertebral subluxation complex (VSC).

### Professional recognition

As an unregulated profession, chiropractic in Australia was, in many respects, the master of its own destiny. However, in 1977 a dramatic change occurred that in many ways changed the future direction of chiropractic in Australia. In that year Edwin Webb and his colleagues compiled and published the "Report of the Committee of Inquiry into Chiropractic, Osteopathy, Homoeopathy and Naturopathy", commonly called "The Webb Report" [5].

This government report paved the way for registration of chiropractors, Australia wide, the establishment of government funded teaching institutions, the beginnings of a research culture and acceptance by mainstream health professions. Webb and his committee recommended that chiropractors and osteopaths be registered but with a proviso: *"The Committee recommends that chiropractic and osteopathy should not be given legal recognition in any form which would imply that they are alternative health systems." *This suggested caveat on the registration of chiropractors and osteopaths in 1977 has equal relevance today, particularly when one considers the road by which the fundamentalists of this profession would have it travel into the future.

The Report also noted: *"Plenty of anecdotal evidence has been published both as to the "cures" produced by chiropractic, osteopathy .....Virtually none of this arises from properly controlled experiments or from the statistical analysis of large samples. Evidence it may be, but not scientific evidence..."*

It is now over 30 years since that report was written, yet the "subluxationists" [6] in the profession still rely on such anecdotal research to support the "Palmer" theory of "dis-ease" [7].

### The cross roads

Chiropractic practice in Australia in the 1970's was much like it is today. Most chiropractors focused on and promoted treatment for musculoskeletal conditions. However, there is today, as there was then, another group of chiropractors, which were indoctrinated with the "true belief" that subluxations were the root of all man's ills.

They were the pseudo religious zealots whose ideology alienated chiropractic from science-based healthcare and prevented it from gaining the level of credibility and cultural authority required to establish itself on equal ground with other mainstream health professions [8]. The "subluxationists" of today appear to be no different and as Keating et al [9] write: *"The dogma of subluxation is perhaps the greatest single barrier to professional development for chiropractors. It skews the practice of the art in directions that bring ridicule from the scientific community and uncertainty among the public. Failure to challenge subluxation dogma perpetuates a marketing tradition that inevitably prompts charges of quackery. Subluxation dogma leads to legal and political strategies that may amount to a house of cards and warp the profession's sense of self and of mission."*

But around 1991 a change was occurring, which commenced with the publication of the RAND report, "The Appropriateness of Spinal Manipulation for Low Back Pain" [10]. This report gave support for the use of manipulation in the treatment of acute low back pain: *"...support is consistent for the use of spinal manipulation as a treatment for patients with acute low back pain and an absence of other signs or symptoms of lower limb nerve root involvement."*

This sound scientific study conducted by an internationally recognised research organisation, and headed by a renowned epidemiologist, showed that spinal manipulation seemed to be effective for acute low back pain. This independent study provided the public, governments, third party payers and mainstream healthcare professions, the proof that spinal manipulation really worked.

Unfortunately, this study was hijacked by an overzealous section of the chiropractic profession, who wrongly broadcast that it was chiropractic, rather than manipulation, that was shown to be beneficial and the authors were forced to publicly admonish this section of the profession by stating: *"Misrepresentation of the RAND study undermines chiropractic's credibility" *and *"Our results have been seriously misrepresented by chiropractors" *[11].

Also around this time a keynote address was given in Melbourne, Australia, at the Chiropractors & Osteopaths Musculoskeletal Interest Group (COMSIG) Conference [1]. This was the first reference to the profession being at a "crossroads" and it was provided by Professor Evan Willis, a sociologist from Latrobe University, Melbourne, whose special interest was in health policies and the social structure of healthcare. Willis concluded: *"So finally which way at the crossroads? If research is the name of the game then chiropractic needs to be in it and urgently so. Patient testimonials have long ceased to be effective in persuading."*

Even then, Willis recognised that the profession could not continue down the same road as it had in the past and expect to prosper, as it was no longer the master of its own destiny. Chiropractic was now becoming more accountable to both government and the public and to survive, inter-professional co-operation was required: *"...chiropractic cannot continue down the same path as previously and expect it to serve them as well has been the case in the past. An increasingly informed consumer orientated public and governments wishing to scrutinize every cent of public expenditure is the name of the game.[sic] Seeking to build bridges with other practitioners interested in musculoskeletal medicine is an important objective." *[1]

### The road to ideology or science

The early 1990's also saw a resurgence of "chiropractic philosophy" in Australia and, with it, the VSC. The source of this philosophical push was the Chiropractors Association of Australia (CAA). Through the CAA's "Core Values" and "Vision Statement" [12], the profession was once again torn between the pursuit of science or the adherence to ideological dogma. Chiropractic fundamentalist "Core Values" (Appendix 1), such as, *"that subluxations compromise the expression of innate intelligence, and that prevention and removal of subluxations will facilitate the expression of optimal health"*, regrettably underpin and dictate the policies and actions of this organisation.

Juxtaposed to this return to ideology, the 1990's saw a push for an evidence-based approach to practice from Australia's mainstream healthcare industry.

It also appeared that the CAA came to a realisation that what was needed was more than just anecdotal evidence to legitimize and support its "Vision" and "Core Values" and therefore amended its research grant guidelines in order to match its "Core Values" and "Vision". Also at this time, the Australian Spinal Research Foundation (ASRF) narrowed and promoted its research grant focus to studies concerned only with the VSC. The Vision Statement of the ASRF [13] states, *"We are the Research Foundation which demonstrates that subluxation-based chiropractic care enhances quality of life and improves human performance." *The ASRF's Research Culture Statement [13] states: *"Our focus is on funding research that investigates the nature of vertebral subluxation and its impact on physiology, health and quality of life."*

However, quality scientific research on spinal manipulation and chiropractic care continued throughout Australia, and internationally, by highly skilled and dedicated chiropractic and other health care researchers [14]. Through this research chiropractic gained an increasing but reserved acceptance and recognition by Australian governments, third party payers, other health disciplines and the public.

Chiropractic achieved this improved standing, not by proclaiming the evils of the ubiquitous VSC and the benefits from removing such an evil entity, nor by relying on the scant and feeble research base supporting this theoretical construct but by good scientific investigation and the publication of high quality research.

The second reference to chiropractic and crossroads came in 2002 when Meeker and Haldeman [2] highlighted the positive changes that had occurred in the profession in the preceding 20 years. They highlighted changes over the past two decades, such as changes in perception from alternative and unorthodoxy to mainstream, both within and outside the profession. Chiropractic was used more than any other complementary and alternative medicine (CAM) group; chiropractic usage in the USA tripled from 3.6% to 11%; patient satisfaction was high; there would be an estimated 100,000 chiropractors in the US by 2010, and, chiropractic care was now included in US Medicare and the majority of private health insurers.

Meeker and Haldeman also discussed how the profession could abandon its alternative tag and become fully integrated into mainstream health care: *"The next decade should determine whether chiropractic maintains the trappings of an alternative healthcare profession or becomes fully integrated into all healthcare systems." *Also, *"In today's dynamic health care milieu, chiropractic stands at the crossroads of mainstream and alternative medicine. Its future role will probably be determined by its commitment to interdisciplinary cooperation and science-based practice*." These are the same crossroads referred to, and the same recommendations, made by Evan Willis, some 10 years earlier [1].

The third reference to chiropractic and crossroads was in 2009 when Ebrall [3] saw the situation quite differently. He saw the crossroads as really a fork in the road, and suggested that our profession drove over the crossroads sometime ago and that they are now, *"but a diminishing dot in our collective rear-view mirror" *and that chiropractic is, *"a dynamic and decidedly unique paradigm of enhanced health and well-being centred on the identification and adjustment of spinal subluxations."*

While it may well be that one group of Australian chiropractors travelled down a fork in the road on their quest to validate the theoretical construct of the VSC, that road has inevitably taken them back to the crossroads and the stark choice of science or ideology.

### Chiropractic today

So what is the state of chiropractic in Australia today? There are over 4300 registered chiropractors in Australia; there are three government funded chiropractic educational programs; chiropractic care is recognised and paid for by government funded workers compensation and transport accident insurers; the cost of chiropractic care is reimbursed by all major private health insurers, and chiropractors are publishing high level research in prestigious national and international journals.

That's the positive, encouraging and illuminating side. But there is a negative, disheartening and dark side.

While Australian data are lacking, the US adult use of chiropractic services declined from 9.9% in 1997 to 7.4% in 2002. This was the largest relative decrease among CAM professions and, as of 2007, only 7% of the US population was being reached by chiropractic [15].

Chiropractic does not have the same level of mainstream credibility as other healthcare professions. Public perception of chiropractic compares unfavourably with mainstream medicine, with regard to ethics and honesty. In a 2006 Gallup Poll of US adults, chiropractors rated last among seven healthcare professions for being very high or high in honesty and ethical standards [16,17].

In 2006, Australian Readers Digest conducted a poll on the most trusted professions [18]. Chiropractors were placed 13th on a list of 30 professions. Placed at 13th, I suppose the profession can console itself in the knowledge that they rated higher in trust than psychics, ministers of religion, car salesmen and politicians but poorer for the knowledge that the public trusts hairdressers more than chiropractors. And while such polls may not be good scientific evidence, they agree with similar polls on the public's perception of chiropractors [19].

The last Newsletter of the Chiropractors Registration Board of Victoria (CRBV), Australia, noted a total of 42 formal complaints against registered chiropractors in Victoria [20]. From personal experience, as a former member of the CRBV, many complaints emanated from the widespread use of false and misleading advertising or advertising designed to engender fear and distress. The use of terms such as, "silent deadly killers" and "spinal decay", when referring to subluxations, do little to promote the profession in a positive light.

### Vertebral Subluxation Complex: use and misuse

What has fuelled this diminishing market share, public distrust and numerous complaints of misconduct? The answer is the road to the VSC. However, it is not this theoretical construct itself that has created this situation and led us back to the crossroads, but rather the way it is taught, sold and promoted, to not only chiropractors but the general public.

In Australia, the "Peak Body" of the chiropractic profession, the CAA, actively promotes subluxation based chiropractic via its previously mentioned "Core Values" (Appendix 1) [12]. Based on the VSC, the CAA's "Vision Statement" [12] envisages chiropractic as a separate and distinct alternative health system: *"To achieve a fundamental paradigm shift in healthcare direction where chiropractic is recognised as the most cost efficient and effective health regime of first choice that is readily accessible to all people*." In light of this "Vision Statement", it is worthwhile remembering the recommendation of the Webb Report, thirty three years ago [5], *"The Committee recommends that chiropractic and osteopathy should not be given legal recognition in any form which would imply that they are alternative health systems."*

Chiropractic trade publications and so-called educational seminar promotion material often abound with advertisements of how practitioners can effectively sell the VSC to an ignorant public. Phrases such as "double your income", "attract new patients" and "keep your patients longer in care", are common enticements for chiropractors to attend technique and practice management seminars. Selling such concepts as lifetime chiropractic care, the use contracts of care, the misuse of diagnostic equipment such as thermography and surface electromyography and the x-raying of every new patient, all contribute to our poor reputation, public distrust and official complaints.

To illustrate how the VSC dogma and ideology can be misused by some chiropractors, it is worth reviewing the case of Dr. Mark Pearson-Gills, who was disciplined by the CRBV for advocating a 60 visit, 12 month treatment plan for a 41/2 week old infant. The defendant later appealed the decision in the Victorian Civil and Administrative Tribunal (VCAT) and the transcript of the proceedings is on the public record [21]. The facts of the case were that *"*Baby CC was approximately 41/2 weeks old, when her mother brought her to see Dr. Pearson-Gills, after reading an advertisement promoting the applicant's practice and offering a first session at a reduced rate of $20. Dr. Pearson-Gills explained in a clinic handout that, *"The SOLE purpose of your chiropractic examination and care is to locate and correct subluxations." *After examining the baby, using thermography and other means, Dr. Pearson-Gills recommended a 60-visit 12-month plan of care.

It is likely that any reasonable person presented with this scenario would find such a plan indefensible and a prima facie case of professional misconduct.

However, Dr. Pearson-Gills produced expert witnesses and several of his peers to defend his actions. The defence rested not on scientific evidence but the philosophical underpinning of finding and correcting subluxations.

In summing up, the Presiding Member of the tribunal discredited the testimony of one expert witness, Dr. Matthew McCoy, from the USA: ***"****Dr McCoy gave evidence at the request of Dr. Pearson-Gills and his solicitor and his attendance in Australia was funded by Dr Pearson-Gills. Despite his protestations that he appeared against chiropractors, the inescapable conclusion is that Dr McCoy is a partisan witness committed to defending the practices of subluxation based chiropractors against malpractice claims and registration board investigation." and ".... I will discount any evidence that Dr McCoy gave, which supported the applicant's case. I gained the impression watching him that he had flown to Australia to give evidence to VCAT as part of his job in supporting WCA (World Chiropractic Alliance) practitioners."*

In my view, Smith's words of 1999 [19] resonate today, with the same clarity: *"Why do we tolerate the charlatans, hucksters, profiteers, and wild-eyed 'philosophers' who taint our profession's image, who obstruct political unity and espouse untrue science that cannot withstand the test of research; who recruit patients with gimmicks, and who mislead naive students and young practitioners with dogma and promises of great wealth? Is it because profession ethics is mostly lacking in chiropractic? Is it due to a laissez faire attitude within chiropractic where anyone can say anything under the guise of "philosophy"? Or is mainstream chiropractic simply scared to confront these fringe elements, fearful of litigation or argument? Have we become a profession ruled by a vocal minority (the Ouiji board practitioners), hellbent on keeping our profession in the past with dogma dominating over science, with leaders who espouse anti-scientific rhetoric, with practitioners who give free spinal exams and $10 office visits, all the while masquerading as "principled" chiropractors who preach unproved health gospel? Is this characterization wrong, or painfully accurate? You tell me."*

For the true believer, the naive practitioner or undergraduate chiropractic student, who accepts in good faith the propaganda and pseudo-science peddled by the VSC teachers, mentors and professional organisations, the result is the same, a sense of belonging and an unshakable and unwavering faith in their ideology.

Others use the propaganda and pseudo-science as a convenient way to justify the exploitation of their patients, and diminish their social and ethical responsibilities as a registered health practitioners, and because their peers are doing the same thing, they reason that they are less accountable; "et to quoque".

### Subluxation: fact and fiction

The CAA and ASRF continue their search for the "holy grail" of evidence to support their subluxation based ideology, but they, and their international counterparts, have failed to produce any worthwhile evidence that subluxations actually exist, let alone adversely impact on a person's health or well-being. Nor have they shown that removal of such an entity has any positive impact on health [9,22,23]. The reality is that the VSC remains a theoretical construct.

Today's culture of evidence based practice has reluctantly led those who promote the VSC, to do so via "research evidence". However, this so-called evidence, is invariably a congealed mix of reality, half truths and "cherry picked quotes" that are misused, misrepresented or overstated. As Phillips [24] writes: "*Chiropractic fundamentalists have sought to make their philosophy more acceptable in today's world by guising scientific rationality under the cloak of emotionality. They selectively incorporate scientific findings that support their "major premise" while shunning or ignoring scientific findings incongruent with their way of thinking"*.

A recent article published in the ASRF Newsletter, known as Illuminate, highlights this point [25]: *"Flu prevention should include chiropractic. Tell your patients why" *and *"Through research we know that chiropractic has beneficial effects on........ pulmonary function and other immune system processes, stated Matthew McCoy, DC, MPH, Editor of the Journal of Pediatric, Maternal & Family Health - Chiropractic." *Dr. McCoy then goes on to quote one study, by *"Patricia Brennan PhD and her team" *[26]. This is the same Matthew McCoy, who is also the Editor of the Journal of Vertebral Subluxation Research and whose testimony was rejected in the aforementioned Pearson-Gills case.

The study by Brenann et al [26], quoted above and a couple of other similar studies, are often cited by the VSC proponents, as proof that correction of VSC's improves immune function. However, this particular study provided no such proof or even a connection between subluxations and immune function. All that the Brennan study showed was that if you push on someone's back hard enough you will get a temporary change in the blood levels of certain biochemical markers.

The changes had nothing to do with subluxations, rather the study identified what happens when you apply different force levels during a manipulative thrust. In other words, you may have well got the same result if you had hit someone in the middle of the back with a piece of 4 × 2 timber. Brennan et al state in the conclusion of their paper, that their results cannot be extrapolated to show improved immune function after spinal manipulation: *"Although it is tempting to speculate on the possible biological significance of our results in terms of the potential for improved host defense against invading microorganisms and in terms of the implications in mild inflammatory processes such as hypomobile joints we believe such speculation to be unjustified by the data presented." *But these facts have not stopped the zealots and others from misrepresenting the results of this study.

Currently the CAA and the ASRF in Australia are promoting "wellness care", which involves the detection and adjustment of VSC's. Last year in ASRF's Newsletter, Illuminate [27], Dr. James Chestnut proclaimed: *".... it is not possible to be well if vertebral subluxation complex is present as a vertebral subluxation complex represents a non-homeostatic state ..... which makes a state of wellness impossible." *So, under the paradigm, published by the CAA, it is easy to make the next step and conclude that everyone needs life time chiropractic care to rid their bodies of subluxations in order to stay healthy. Once again, no evidence just belief and rhetoric.

### Chiropractic education

There are currently three undergraduate chiropractic teaching institutions in Australia. All are government funded and part of Australia's mainstream tertiary education system.

The undergraduate chiropractic program at RMIT University [23] promotes the subluxation myth by defining its model of chiropractic to include: *"Functional derangement of the spine and other articulations may occur; where spinal we call this a vertebral subluxation complex (VSC)." *and *"Such derangements may affect the functioning of the neurological system in a variety of ways through a variety of mechanisms; we see such altered function as ranging from the Newtonian and quantifiable findings of pain and altered sensation to the Quantum and qualitative findings of altered cognitive and affective dimensions;" *The author suggests that such existential rhetoric is more suited to a degree in philosophy than a degree in science.

In contrast, in the United Kingdom (UK), the General Chiropractic Council (GCC), a regulatory authority similar to the Chiropractic Board of Australia (CBA), recently sought position statements on the VSC, from the three chiropractic teaching institutions in the UK. Unlike Australia, not one of these institutions taught the VSC theory in the context of modern health care delivery [23].

Associate Professor Phillip Ebrall, Head of the Chiropractic Unit, at RMIT University, has also written extensively about the VSC and, in particular, how it should be taught to chiropractic students [28]. He writes: *"lnspired by a visit to Disneyland this paper explores the challenges associated with the need to teach something that may not exist. It reports lessons learned by viewing a successful commercial illusion that has capacity to inform a pedagogical approach to abstract objects."*

What is the point of teaching something that does not exist? Could it be that the VSC itself is just "successful commercial illusion"?

To illustrate the belief in the VSC and chiropractic fundamentalism, at its extreme, the founder and former President of the largest college of chiropractic in the world was once quoted as saying, *"Rigor mortis is the only thing we can't help!" *[29]. It comes as no surprise that this university is now the subject of a class action law suit, by former students, which alleges breach of contract, for the failure of the university to teach differential diagnosis [30].

Murphy in his paper comparing and contrasting the evolution of chiropractic to the acceptance and integration of podiatry into mainstream healthcare wrote about the teaching of subluxation [31]: *"These concepts are lacking in a scientific foundation and should not be permitted to be taught at our chiropractic institutions as part of the standard curriculum." *and *"Faculty members who hold to and teach these belief systems should be replaced by instructors who are knowledgeable in the evidence-based approach to spine care..."*

The irony of this fervent belief in the VSC and chiropractic philosophy is that its development was not founded on vitalistic theory but rather as a legal strategy, conjured up by an attorney, in the defence of a chiropractor charged with practicing medicine [7,32,33]: *"Many in chiropractic never learned the origin of the pseudo-region or chiropractic philosophy. It was nothing more than a legal tactic used in the Morriubo's case." *[34], and *"B.J. Palmer probably developed his dis*-*ease theory as a result of the winning strategy used by his attorney Thomas Morris to defend Japanese chiropractor Shegatoro Morijubo in Wisconsin in 1907" *[35].

### Back to the crossroads

So where to from here, which road will the chiropractic profession take, subluxation or science?

If we take the path of the VSC, then I have no doubt that whatever acceptance, credibility and privileges the profession has gained in the last 35 years will be rapidly lost.

Chiropractors will be further alienated from mainstream healthcare. The profession will survive but it will again be dumped into the unscientific quack bin with homeopathy and iridology. Others within mainstream healthcare, who are trained in spinal manipulation, will continue to progress and fill the void.

As Nelson et al [36] rightly put it, we have an obligation as licensed health care providers not to promote unscientifically unreasonable beliefs as clinical truths: *"Neither a chiropractor nor any other healthcare provider practicing under the protection of a licensed profession has the ethical right to promote unscientifically unreasonable beliefs. The principle of fidelity and the state of scientific knowledge regarding certain historical chiropractic beliefs should not allow the expression of these beliefs to the patient as clinical truths."*

Further, on subluxation they write, *"A number of models are impractical, implausible or even indefensible from a purely scientific point of view (e.g., subluxation- based healthcare), from a professional practice perspective (e.g., the primary care model), or simply from common sense (e.g. Innate Intelligence as an operational system for influencing health)."*

If we take the road of science, we may still have a chance to establish ourselves as spine care specialists. Murphy [31] writes: *"We see a tremendous opportunity for chiropractic medicine to become what it can and should be: a profession of non-surgical spine specialists who not only offer one useful modality of treatment for spinal pain (manipulation), but offer something much greater and more important - expertise in the diagnosis and management of spinal pain patients."*

A recent decision in the UK by the GCC, may provide us with a road map [23]. A position statement published in early 2010 states:

• *"The chiropractic vertebral subluxation complex is an historical concept but it remains a theoretical model*.

• *It is not supported by any clinical research evidence that would allow claims to be made that it is the cause of disease or health concerns*.

• *Chiropractors are reminded that they must make sure their own beliefs and values do not prejudice the patients' care*.

• *They must provide evidence based care, which is clinical practice that incorporates the best available evidence from research, the preferences of the patient and the expertise of practitioners, including the individual chiropractor her/himself."*

This position statement was also endorsed by the British Chiropractic Association (BCA) [37], the equivalent body in the UK, to the CAA in Australia:

• *"The BCA welcomes today's statement from the General Chiropractic Council (GCC) on Vertebral Subluxation Complex*.

• *For many years, the BCA has not supported the concept of the Vertebral Subluxation Complex*.

• *To facilitate the integration of chiropractic, unsubstantiated historical concepts and ambiguous terminology must be discarded in favour of an emphasis on delivering an evidence-based care model that is easily understood by other members of the healthcare team"*

The decision by the GCC and the BCA followed the failed court case of Simon Singh, a science writer, who was sued for libel by the BCA for claiming that chiropractors used bogus treatments [38].

As a consequence of this libel suit Singh's supporters made complaints to the GCC and the UK's Advertising Standards Authority [29], over the use of the term subluxation and the unsubstantiated claims, made by some chiropractors, of the successful treatment of various diseases such as colic, asthma, irritable bowel syndrome etc., that were purportedly caused by subluxations. These complaints and the subsequent investigations, have led some chiropractic organisations to advise their members to remove references to subluxation and the treatment of a number of diseases in their advertising [39].

There are also some parallels between the GCC position statement and the recent release of the Code of Conduct for Chiropractors, by the newly formed CBA [40]. The CBA's Code of Conduct defines good care as, involving evidence informed care, and that chiropractors should avoid expressing their personal beliefs to patients in ways that exploit their vulnerability or that are likely to cause them distress.

In response to the GCC's and CBA's positions, it would appear that the true believers and others are attempting to marshal their forces. At the forefront is Dr. McCoy whose article, *"The death of subluxation."*, published in the ASRF Newsletter, Illuminate, [41]: encourages like minded chiropractors to, *"Pick up your pitch forks and torches and get ready to storm the castle" *and *"Subluxation, family wellness focused chiropractors need to take over the regulatory boards, agencies and organisations in this profession."*

## Summary

So it would appear, that for the past thirty odd years all we have done is gone around in circles and we are once again back at the crossroads," Science or Subluxation."

The "subluxationists" see chiropractic as a unique system of healthcare supported not by science, but an ideology and pseudo-science, yet they still want to be accepted into mainstream healthcare and enjoy all the benefits that come with the acceptance and credibility of a science based discipline. If this profession is to move forward it has to base its future on science and not ideological dogma.

In a recent commentary, Scott Haldeman [42] highlighted the need to base therapy on evidence: "*It does not serve patients to provide treatment that has been shown to be ineffective or where there is insufficient evidence to reach a conclusion when there are other options available that have been demonstrated to be beneficial." *and *"It is not acceptable today to claim that a treatment is effective in helping patients when there is no evidence to support these claims."*

Finally, Haldeman concludes: *"It does not help the reputation of a profession that is striving to be considered the authority in a field, if practitioners are unwilling to understand and practice according to the latest clinical evidence"*.

Hopefully, Dr McCoy is right and the "subluxation" will die or at least assume a more credible identity. But similar to what happened in the UK, it may require clear and decisive action on the part of the CBA and/or the Australian Competition and Consumer Commission (ACCC) to finally put it to rest.

However, I also think it is time for the moderates and conservatives within our profession to become more involved with shaping its future. For too long now the "the near empty tin has made far too much noise". It is about time government, other health care providers and the public were made aware that not all chiropractors are pseudo religious zealots who have abandoned science for ideology: *"Chiropractic does have a large silent majority which seems content to tolerate the fringe elements and to let other people fight their political battles, allthewhile wondering why our situation doesn't seem to be improving." *[19].

Maybe it's time for the silent majority to pick up their pitch forks and torches, and get ready to storm the castle, and to take over the regulatory boards, agencies and organisations of this profession?

## Competing interests

The author declares that they have no competing interests.

## Appendix 1

### Chiropractors Association of Australia

Core Values

***We recognise***...

*• and respect a universal intelligence (or order) in all matter and an innate intelligence within a living organism that strives to preserve life and, if uninhibited, will express optimal well being*.

*• that the practice of chiropractic focuses on the relationship between structure (primarily the spine) and function (as coordinated by the nervous system) and how that relationship affects the preservation and restoration of health*.

*• that subluxations compromise the expression of innate intelligence, and that prevention and removal of subluxations will facilitate the expression of optimal health*.

*We respect, care about and are committed to the individual's holistic well being and emphasise the inherent recuperative power of the body to heal itself without the use of drugs or surgery and value the importance of intellectual honesty, scientific and academic excellence and the maintenance of integrity in serving the individual, the community and the profession*.

### Vision Statement

*To achieve a fundamental paradigm shift in healthcare direction where chiropractic is recognised as the most cost efficient and effective health regime of first choice that is readily accessible to all people*.
